# The antibacterial activity and mechanism of a novel peptide MR-22 against multidrug-resistant *Escherichia coli*

**DOI:** 10.3389/fcimb.2024.1334378

**Published:** 2024-01-24

**Authors:** Chunren Tian, Na Zhao, Longbing Yang, Fei Lin, Ruxia Cai, Yong Zhang, Jian Peng, Guo Guo

**Affiliations:** ^1^ School of Basic Medical Sciences, The Key and Characteristic Laboratory of Modern Pathogen Biology, Guizhou Medical University, Guiyang, China; ^2^ Clinical Laboratory, Guiyang Hospital of Guizhou Aviation Industry Group, Guiyang, China; ^3^ Key Laboratory of Environmental Pollution Monitoring and Disease Control, Ministry of Education, Guizhou Medical University, Guiyang, China; ^4^ Translational Medicine Research Center, Guizhou Medical University, Guiyang, China

**Keywords:** MR-22, antimicrobial peptide, *Escherichia coli*, multidrug-resistant, antimicrobial mechanism

## Abstract

**Introduction:**

Bacterial infections have become serious threats to human health, and the excessive use of antibiotics has led to the emergence of multidrug-resistant (MDR) bacteria. *E. coli* is a human bacterial pathogen, which can cause severe infectious. Antimicrobial peptides are considered the most promising alternative to traditional antibiotics.

**Materials and methods:**

The minimum inhibitory concentration (MIC), minimum bactericidal concentration (MBC) and hemolytic activity were determined by the microdilution method. The antimicrobial kinetics of MR-22 against *E. coli* were studied by growth curves and time-killing curves. The cytotoxicity of MR-22 was detected by the CCK-8 assay. The antimicrobial activity of MR-22 in salt, serum, heat and trypsin was determined by the microdilution method. The antimicrobial mechanism of MR-22 against drug-resistant *E. coli* was studied by Scanning Electron Microscope, laser confocal microscopy, and Flow Cytometry. The *in vivo* antibacterial activity of MR-22 was evaluated by the mice model of peritonitis.

**Results and discussion:**

In this study, MR-22 is a new antimicrobial peptide with good activity that has demonstrated against MDR *E. coli*. The antimicrobial activity of MR-22 exhibited stability under conditions of high temperature, 10% FBS, and Ca^2+^. However, a decline of the activity was observed in the presence of Na^+^, serum, and trypsin. MR-22 had no significant cytotoxicity or hemolysis *in vitro*. SEM and fluorescent images revealed that MR-22 could disrupt the integrity of cell membrane. DCFH-DA indicated that MR-22 increased the content of reactive oxygen species, while it decreased the content of intracellular ATP. In mice model of peritonitis, MR-22 exhibited potent antibacterial activity *in vivo*. These results indicated that MR-22 is a potential drug candidate against drug-resistant *E. coli*.

## 1 Introduction

*Escherichia coli* (*E. coli*) is a common Gram-negative bacterial species in the intestinal tract of both humans and animals. *E. coli* can cause severe infectious if it invades the bloodstream, urinary tract, abdominal cavity, or other areas through a wound (Paitan, 2018). Antibiotics are effective therapeutic drugs in clinic. However, with the overusing antibiotics, drug-resistant bacterial have emerged, and even MDR also emerged (Frost et al., 2019; de Kraker and Lipsitch, 2022). MDR denotes the occurrence where microorganisms exhibit insensitivity or resistance to antibiotics characterized by distinct molecular targets (Ren et al., 2022). These bacteria exhibit high levels of resistance and tolerance to antibiotics by increasing their own resistance mechanisms, producing extended spectrum β-lactamases that hydrolyze penicillin and cephalosporin drugs to render antibiotics ineffective, or acquiring resistant genes to avoid being suppressed or killed by antibiotics (Breijyeh et al., 2020). Consequently, treating MDR bacteria poses a formidable challenge within the realm of conventional antibiotic therapy. Presently, the primary approach for addressing bacterial resistance during the course of treatment involves either the synergistic deployment of traditional antibiotics or the utilization of the latest generation of antimicrobial agents (Bassetti and Peghin, 2020). For instance, colistin, carbapenems, and carbapenem derivatives were frequently employed in the therapeutic management of infections arising from drug-resistant bacterial strains (Karaiskos et al., 2019). Inaddition, the third- or fourth-generation cephalosporins represent the prevailing choice for the multi-drug resistant Gram-negative infections (Corona et al., 2023). Polymyxin and tigecycline are regarded as viable alternatives against MDR Gram-negative bacterial. However, it is noteworthy that resistance to both classes of antibiotics is on the rise (D'Onofrio et al., 2020; Fu et al., 2021; Udeani and Ugah, 2021). Therefore, the development of novel antibacterial drugs that are less susceptible to resistance mechanisms and resistant gene mutations than traditional antibiotics is crucial for treating bacterial infections.

Antimicrobial peptides (AMPs), as small proteins with broad-spectrum antimicrobial activity, are promising candidates to against drug-resistance bacteria (Browne et al., 2020). Studies have shown that peptides extracted from amphibians have been effectively used to treat local infections caused by various drug-resistant strains, including local infections caused by *E. coli* infection (Marani et al., 2017). Natural AMPs offer the advantage of a short killing time and broad-spectrum antibacterial activity, with effective killing properties on bacteria, fungi, and viruses (He et al., 2018; Buda De Cesare et al., 2020; Neshani et al., 2020). At the same time, emerging types and technologies of AMPs, such as artificially synthesized multifunctional peptides, cell-penetrating peptides, and peptide-drug conjugates, are broadening the clinical applications of peptides as a therapeutic option for treating diseases (Gao et al., 2018; Boparai and Sharma, 2020). These advantages enable them to hold great promise as the most potential alternatives to conventional antibiotics. Nevertheless, their high toxic and hemolytic properties limit their practical applications (Oddo and Hansen, 2017; Yang et al., 2023). Due to the diversity of antimicrobial mechanisms, the mechanism of antimicrobial peptides is still unclear.

Using the novel theory of multitask adaptive modeling, we established a model for antimicrobial peptides and identified several potent antimicrobial peptides from the UniProt (Zhang et al., 2022). In the screening of antibacterial spectrum, MR-22 not only showed a variety of anti Gram-negative bacteria activities, but also had prominent antibacterial activity against drug-resistance *E. coli*. To evaluate the antibacterial activity and mechanisms of MR-22, we conducted experimental verification on *E. coli* ATCC 25922 and clinically MDR isolated E19. The results investigated the antibacterial mechanism of MR-22 on *E. coli* ATCC 25922 and E19 at the physicochemical and morphological, evaluated the *in vivo* efficacy through animal experiments. The findings of this study provide a theoretical basis for the development of novel antimicrobial drugs research and development.

## 2 Materials and methods

### 2.1 Antimicrobial peptide synthesis and validation

MR-22 (MAKRRKKAKKKAKKAKKRRRRR-NH_2_) was synthesized by solid phase synthesis by GL Biochem (Shanghai, China). The MR-22 was purified to greater than 95% by reversed phasc-high performance liquid chromatography (RP-HPLC) (**Supplementary Figure 1A**). The molecular weight was determined by high performance liquid chromatography-tandem mass spectrometry (HPLC/MS/MS) (**Supplementary Figure 1B**). MR-22 was prepared in water at a final concentration of 1 mg/mL. The hydrophobicity of MR-22 was analyzed using the online software HeliQuest (https://heliquest.ipmc.cnrs.fr/). The 3D spatial structure of MR-22 was predicted via the I-TASSER (https://zhanggroup.org/I-TASSER/) (Yang and Zhang, 2015; Zhang et al., 2017; Zheng et al., 2021).

### 2.2 Bacterial strains and growth medium

The clinical strains used in this study were obtained from a tertiary healthcare facility located in Guiyang, a city situated within the Guizhou Province of China. The *E. coli* ATCC 25922 was obtained from the Key and Characteristic Laboratory of Modern Pathogen Biology at Guizhou Medical University in Guiyang, China. All strains were stored at –80°C in LB medium and routinely cultured at LB medium at 37°C.

### 2.3 Antibacterial activities assay

#### 2.3.1 Determination of minimum inhibitory concentration (MIC) and minimum bactericidal concentration (MBC)

The MIC were determined by a micro dilution method in Mueller-Hinton broth (MHB) (Cuenca-Estrella et al., 2010). The final concentration of bacterial suspension in MHB were 1.0×10^6^ CFU/mL. The final concentration of MR-22 ranged 1 ~ 256 μg/mL in 96 wells plate broth at 37°C for 16~18 h. Imipenem was employed as positive control, bacterial grown without treatment as negative control and MHB as blank control. MIC determination was performed by reading the OD_600_ nm. Take 100 μL samples in holes that have never seen bacterial growth and place them on the LB agar board. The concentration of MR-22 with no bacterial growth was defined as the MBC.

#### 2.3.2 Growth curve assay

The determination of the growth curve of *E. coli* in response to MR-22 was performed similar to the method described by (Rasool et al., 2016). The bacterial suspensions of *E. coli* ATCC 25922 and E19 were resuspended to 1.0×10^6^ CFU/mL with different concentrations of MR-22 added (the final peptide concentrations of 1, 2, 4 ×MIC) or PBS in 96-well plates and cultured at 37°C. The absorbance at 600 nm was measured by using a microplate reader for consecutive 24 h at 2 h interval.

#### 2.3.3 Time-kill assays

The time-kill kinetics of MR-22 on *E. coli* were determined following the protocol described by (Lu et al., 2022). The bacterial suspensions (1.0×10^6^ CFU/mL) were mixed with different concentrations of MR-22, then cultured at 37°C. The final concentrations of MR-22 ranged 1, 2, 4 ×MIC. At 0, 1, 2, 4, 6, 12, and 24 h, 10 μL aliquot samples were diluted in 90 μL PBS and 5 μL aliquot of the dilutions was spread onto LB agar plates. After incubation at 37°C for 24 h, the colony counts were determined and the time-killing curve was plotted.

### 2.4 Cytotoxicity assay

Cell Counting Kit-8 assay (CCK-8) is a premixed, readily applicable colorimetric assay used for determining the number of viable cells in a sample. The cytotoxicity of MR-22 on HK-2 and L-O2 were performed according to the methods of (Chen et al., 2022) with some modifications. Briefly, HK-2 cells or LO-2 cells were cultured in Dulbecco"s Modified Eagle Medium (DMEM) containing 10% fetal bovine serum (FBS) and 1% Penicillin-streptomycin at 37°C with 5% CO_2_. All cells were inoculated into 96-well plates at a density of 1×10^6^ cells/well for 24 h, and then treated with MR-22 (1 ~ 256 μg/mL) for 5 min or 24 h. Cells were cultured without MR-22 in DMEM medium were used as a negative control, and DMEM only was used as a blank control. After the incubation, 10 μL of CCK-8 solution was added to each well and incubated for 2 h. The absorbance was measured at 450 nm with microplate reader.


$$\text{The relative growth rate (\%)=}\frac{{\text{(A}}_{450 nm}{\text{ of test well-A}}_{450 nm}\text{ of blank control)}}{{\text{(A}}_{450 nm}{\text{ of negative control- A}}_{450 nm}\text{ of blank control)}}x100\%$$

### 2.5 Hemolytic activity assay

The hemolytic activity of MR-22 on human red blood cells was assessed according to a previous study (Zhou et al., 2021). Human blood cells were collected by centrifugation at 1000 g for 10 min, then washed three times with 1×PBS. Collected red blood cells were suspended in 1× PBS to 4%. Then, 100 μL 4% red blood cell suspension was added to different concentration of MR-22 (1 ~ 256 μg/mL) in 96-well plates and incubated for 1 h at 37°C. The absorbance at 540 nm was measured with microplate reader. While PBS served as the negative control, a blood cell suspension treated with 1% Triton X-100 was used as a positive control for 100% hemolysis.


$$\text{The hemolysis rate (\%)}=\frac{({\text{A}}_{540\text{ nm}}{\text{ of test well- A}}_{540\text{ nm}}\text{ of negative control)}}{\left({\text{A}}_{540\text{ nm}}{\text{ of positive control - A}}_{540\text{ nm}}\text{ of negative control}\right)}\times 100\%$$

### 2.6 Antimicrobial activity in the presence of salts, serum, temperature and trypsin

In order to study the activity of MR-22 in high-salt concentration, mice serum, different temperatures or trypsin, MIC once again conducted tests as described above.

*E. coli* ATCC 25922 and E19 were incubated in different concentrations MR-22 in MHB with 150 mM NaCl, 2 mM CaCl_2_, 10% mice serum and 10% FBS in 96-well plates and cultured at 37°C for 16 h. The resulting solutions were subjected to MIC testing to evaluate any alterations in the antimicrobial activity of MR-22.

To assess the thermal stability of MR-22, it was subjected to various temperatures (40°C, 60°C, 80°C, and 100°C) for 30 min. Then, reducing to room temperature, and the MIC was subsequently determined.

MR-22 was mixed with a solution containing 1 μg/ml of trypsin and incubated at 37°C for 2 to 12h. Then, trypsin inactivation was carried out at 60°C for 30 min. The antimicrobial activity of the samples was tested to assess the activity of MR-22 and its analogs after the treatment by trypsin. The untreated MR-22 served as the control group for comparative analysis.

### 2.7 Scanning electron microscope observation

To investigate the morphological changes induced by MR-22 on *E. coli*, bacterial suspensions (1.0×10^6^ CFU/mL) was treated with MR-22 at final concentrations of 1 × MIC or PBS for 2 h. The bacterial were washed twice with PBS, fixed with 2.5% of glutaraldehyde at 4°C overnight. Then, the bacterial were dehydrated in a series of different concentrations of ethanol solutions. A bacterial suspension without MR-22 was used as a control for comparison purposes. The image acquisition was performed using a Hitachi Regulus SU8100 (Tokyo, Japan).

### 2.8 Bacterial viability

The fluorescence probe staining method and confocal laser scanning microscopy (CLSM; Olympus SpinSR10, Japan) were used to visualize the proportion of viable and dead bacteria (Zapata et al., 2008). The bacterial suspension (1.0 × 10^6^ CFU/ml) and the MR-22 of different concentrations incubated for 1h at 37°C, centrifugating at 2,000 *g* for 5 min collects bacteria. The bacterial were washed twice and resuspended in PBS. Next, the final concentrations of 10 μM SYTO 9 and 10 μM PI were added to each group and incubated in the dark at 37°C for 15 min. After completion of the incubation, unbound fluorescent dye was washed away with PBS. The fluorescent images of samples were analyzed using CLSM.

### 2.9 Membrane permeability assay

According to the previously described method (Fadhel Abbas Albaayit et al., 2022), bacterial suspensions were diluted with PBS to concentration of 1.0×10^6^ CFU/mL. The bacterial suspensions were incubated in different concentrations of (the final peptide concentrations of 1, 2, 4 ×MIC) MR-22 or PBS in Eppendorf tubes and cultured at 37°C for 2 h. 10 μM of propidium iodide (PI; Sigma, US) was added to each group and incubated at 37°C for 15 min in the dark. The samples were analyzed using a CytoFLEX flow cytometry (Beckman Coulter, USA), and the positive rate of PI was utilized as an indicator of bacterial membrane permeability.

### 2.10 Reactive oxygen species measurements

The fluorescence probe 2’,7’-dichlorodihydrofluorescein diacetate (DCFH-DA) was used to measure the intracellular generation of ROS. The bacterial suspension (1.0 × 10^6^ CFU/mL) were mixed with DCFH-DA (the final concentration of 10 μM) and incubated for at 37°C 30 min in the dark. After incubation, the suspensions were treated with MR-22 (1, 2, 4 × MIC) at the indicated concentrations for 1 h at 37°C. The fluorescence intensity was evaluated using a multi-function fluorescent enzyme marker with an excitation wavelength of 488 nm and an emission wavelength of 525 nm. N-acetylcysteine (NAC) at a concentration of 24 mM was employed as a control agent for the purpose of quenching the production of reactive oxygen species.

### 2.11 ATP determination

The MR-22 solution was added to bacterial suspensions (1.0 × 10^6^ CFU/mL) at different final concentrations (1, 2, 4 × MIC) incubated at 37°C for 1 h. The mixture was subsequently centrifuged and the resulting supernatants were collected. Finally, the intracellular ATP concentration was determined using an ATP Assay Kit (Beyotime, China) according to the manufacturer’s instruction.

### 2.12 Mice infection model

This study utilized female C57BL/6J mice aged 6-8 weeks, weight range of 18-20 g, which were obtained from SPF Biotechnology Co., Ltd (Beijing, China). The animals were housed under maintained on a standard 12 h light/12 h dark cycle. All animal experiments were conducted in accordance with the Ethical Principles in Animal Research adopted by Guizhou Medical University and were approved by the Institutional Animal Care and Use Committee.

To evaluate the *in vivo* efficacy of MR-22, we used a mice peritonitis model. A total of 60 mice were divided into three groups: treatment group (10 mg/kg MR-22, intraperitoneal injection), positive control group (10 mg/kg imipenem, intraperitoneal injection), and normal control group (PBS, intraperitoneal injection). Peritonitis was induced by intraperitoneal injection of 1.0 × 10^8^ CFU bacterial inoculum in 0.2 mL of *E. coli* E19. Survival rates were recorded every 12 h for 7 days.

To study the protective effects of MR-22, 10 mice in each group were sacrificed, and peritoneal lavage fluid, blood, liver, and kidney were collected 36 h after administration. The number of colonies in peritoneal lavage fluid and blood were determined by culturing on LB agar, while the tissues were fixed in 4% paraformaldehyde and subjected to hematoxylin and eosin staining to study the morphology.

### 2.13 Statistical analysis

All experiments were repeated at least three times. Differences between groups were assessed using Student’s t-test and one-way analysis of variance (ANOVA). Data were expressed as means ± standard deviations (Mean ± SD). *P<* 0.05 was considered as statistical significance.

## 3 Results

### 3.1 Characterizations of antimicrobial peptide

**Figure 1:**
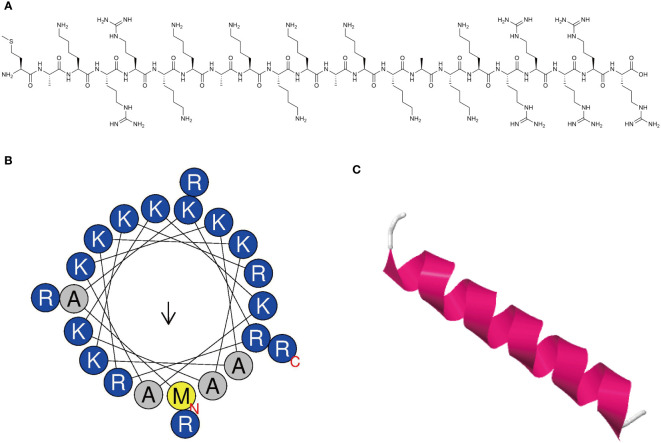
Characterization of novel antimicrobial peptide. **(A)** Chemical structure of antimicrobial peptide. **(B)** Helical wheel projection diagrams of MR-22 using HeliQuest analysis (https://heliquest.ipmc.cnrs.fr/). Hydrophilic amino acids are shown in blue and are positively charged. Amino acids are shown in yellow and gray are hydrophobic. **(C)** Three-dimensional structure of peptide was predicted via the I-TASSER (https://zhanggroup.org/I-TASSER/). The secondary structure is represented by different colors. Magenta represents the helix structure, and white represents the coil structure.

The synthesized peptide and its sequences and biochemical parameters of MR-22 are listed in **Table 1**. MR-22 is a cationic peptide with a purity greater than 95%. The calculated molecular weight was found to be in agreement with the detected molecular weight, indicating accurate synthesis of the peptide. Furthermore, MR-22 is a cationic antimicrobial peptide with a structure mainly composed of helix and coil conformations (**Figure 1**).

**Table 1:** Physicochemical parameters of MR-22.

Peptide	Sequence (N→C)	Formula	Charge*^a^*	pI*^a^*	Calcd MW*^a^*	Obsd MW*^b^*	Purity (%)
MR-22	MAKRRKKAKKKAKKAKKRRRRR-NH_2_	C_119_H_235_N_53_O_23_S_1_	+17	12.79	2808.58	2807.62	95.44

^a^Charge, isoelectric point (pI) and molecular weight (MW) were calculated online at https://web.expasy.org/protparam/. ^b^The observed molecular weight (MW) was determined by liquid chromatograph mass spectrometer (LC-MS).

### 3.2 Antimicrobial activity

*E. coli* ATCC 25922 and clinically drug resistant isolated strains were used as test strains for MIC and MBC assays to investigate the antimicrobial activity of MR-22 (**Table 2**). The of MR-22 MICs ranged from 4 ~ 32 μg/mL. The MICs of sulfamethoxazole tablets, tetracycline, amoxicillin/clavulanic acid, cefepime, levofloxacin, and ciprofloxacin against the sensitive strain were uniformly low, ranging from 0.25 ~ 20 μg/mL. However, these MIC showed varying degrees of increase when drug-resistant bacterial strains tested. The positive control drug is imipenem, which exhibited notable antibacterial efficacy against all strains, with MIC values consistently ≤1 μg/mL. In summary, MR-22’s MBC is equal to MIC.

**Table 2:** Antimicrobial activity of MR-22 against *E. coli*.

*S*trains	MIC (μg/mL)								MBC (μg/mL)
	MR-22	SXT	TET	AMC	FEP	LEV	CIP	IPM	MR-22
ATCC 25922	16	≤20 (S)	≤0.5 (S)	≤4 (S)	≤2 (S)	≤0.5 (S)	≤0.25 (S)	≤1 (S)	16
E01	16	≤20 (S)	≤0.5 (S)	4 (S)	≤2 (S)	≤0.5 (S)	≤0.25 (S)	≤1 (S)	16
E02	16	>320 (R)	>8 (R)	4 (S)	>16 (R)	≤0.5 (S)	≤0.25 (S)	≤1 (S)	16
E03	16	≤20 (S)	>8 (R)	4 (S)	16 (R)	8 (R)	>2 (R)	≤1 (S)	16
E04	8	>320 (R)	>8 (R)	4 (S)	>16 (R)	>8 (R)	>2 (R)	≤1 (S)	8
E05	16	≤20 (S)	>8 (R)	4 (S)	>16 (R)	≤0.5 (S)	≤0.25 (S)	≤1 (S)	16
E06	8	≤20 (S)	>8 (R)	8 (S)	>16 (R)	8 (R)	>2 (R)	≤1 (S)	8
E07	16	>320 (R)	>8 (R)	8 (S)	>16 (R)	≤0.5 (S)	≤0.25 (S)	≤1 (S)	16
E08	8	>320 (R)	>8 (R)	16 (I)	≤2 (S)	≤0.5 (S)	≤0.25 (S)	≤1 (S)	8
E09	16	≤20 (S)	>8 (R)	8 (S)	≤2 (S)	8 (R)	>2 (R)	≤1 (S)	16
E10	16	≤20 (S)	≤0.5 (S)	8 (S)	16 (R)	≤0.5 (S)	≤0.25 (S)	≤1 (S)	16
E11	16	>320 (R)	>8 (R)	≤4 (S)	>16 (R)	≤0.5 (S)	≤0.25 (S)	≤1 (S)	16
E12	16	≤20 (S)	>8 (R)	≤4 (S)	4 (I)	>8 (R)	>2 (R)	≤1 (S)	16
E13	16	≤20 (S)	≤0.5 (S)	16 (I)	≤2 (S)	≤0.5 (S)	≤0.25 (S)	≤1 (S)	16
E14	16	>320 (R)	>8 (R)	≤4 (I)	16 (R)	>8 (R)	>2 (R)	≤1 (S)	16
E15	4	≤20 (S)	≤0.5 (S)	16 (I)	≤2 (S)	0.5 (S)	≤0.25 (S)	≤1 (S)	4
E16	16	>320 (R)	≤0.5 (S)	≥32 (R)	≤2 (S)	1 (I)	0.5 (I)	≤1 (S)	16
E17	32	>320 (R)	≤0.5 (S)	8 (S)	≤2 (S)	>8 (R)	>2 (R)	≤1 (S)	32
E18	8	>320 (R)	≤0.5 (S)	16 (I)	16 (R)	>8 (R)	>2 (R)	≤1 (S)	8
E19	32	>320 (R)	≤0.5 (S)	8 (S)	16 (R)	>8 (R)	>2 (R)	≤1 (S)	32
E20	8	≤20 (S)	≤0.5 (S)	4 (S)	16 (R)	>8 (R)	>2 (R)	≤1 (S)	8
E21	8	>320 (R)	≤0.5 (S)	4 (S)	16 (R)	≤0.5 (S)	≤0.25 (S)	≤1 (S)	8
E22	16	>320 (R)	≤0.5 (S)	16 (I)	≥32 (R)	>8 (R)	>2 (R)	≤1 (S)	16

SXT, compound sulfamethoxazole tablets; TET, tetracycline; AMC, amoxicillin/clavulanic acid; FEP, cefepime; LEV, levofloxacin; CIP, ciprofloxacin; IPM, imipenem; I, intermediate; S, susceptible; R, resistant.

### 3.3 Growth assay and rapid bactericidal efficiency of MR-22 against *E. coli.*

**Figure 2:**
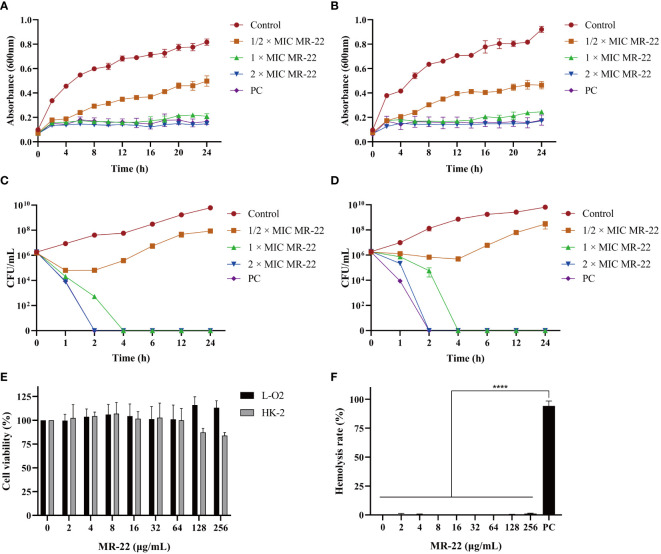
Growth curves of MR-22 against *E. coli* ATCC 25922 **(A)** and E19 **(B)**. Killing kinetics with *E. coli* ATCC 25922 **(C)** and E19 **(D)**. Different concentrations of MR-22 were incubated with approximately 1x10^6^ CFU/ml inoculum. Samples were taken at different time intervals, plated in LB medium and CFUs counted by triplicate. **(E)** Cytotoxicity of MR-22 against L-O2 cells and HK-2 cells determined by CCK-8 assay. **(F)** Hemolytic activities of MR-22 at different concentrations after incubation with 4% human red blood cells for 1 h. Data are presented as the mean ± standard deviation of three independent experiments. ^****^ *P*< 0.0001 compared to the positive control (PC) group. The error bars indicate the mean ± standard deviation (n = 3).

To better understand the action of MR-22 on clinical drug-resistant isolates, the most recalcitrant E19 and non-resistant ATCC 25922 strains were chosen. The growth curves of *E. coli* E19 and ATCC 25922 in the presence of MR-22 were plotted (**Figures 2A, B**). MR-22 dose-dependently inhibited the bacterial growth at concentrations ranging 1 × MIC to 2 × MIC. The kinetic-killing effect of MR-22 against *E. coli* E19 and ATCC 25922 was further evaluated (**Figures 2C, D**). A similar trend was observed in the treatment of *E. coli* E19 and ATCC 25922, eliminating the bacteria within 2 h at concentrations of 1 × MIC and 2 × MIC, respectively. These results demonstrated that MR-22 displayed rapid bactericidal activity against bacterial pathogens.

### 3.4 Effects of salt, serum, heat and trypsin on MR-22 activity

MIC assays were performed to determine the effects of salt, serum, heat, and trypsin on MR-22 activity. The results shown in **Table 3**. The MIC for MR-22 was not changed in the absence of physiological CaCl_2_ compared to control, but in the presence of physiological NaCl were changed to 256 μg/mL. In addition, culture media containing 10% FBS and mice serum were used to simulate *in vivo* matrix environment. The results showed that the antibacterial activity of MR-22 in 10% mice serum reduced only four folds. After pre-incubation in 10% fetal bovine serum, MR-22’s MIC showed no change. Despite alterations in the MICs of MR-22, its antimicrobial efficacy or activity persisted.

**Table 3:** Stability of MR-22 against *E. coli*.

Treatments	MR-22(MIC, μg/mL)	
	*E. coli* ATCC 25922	*E. coli* E19
Control	16	32
150 μM Na^+^	256	256
2 mM Ca^2+^	8	8
10% FBS	16	32
10% Serum	64	128
1 μg/mL Trypsin	>256	>256
40°C 30 min	16	32
60°C 30 min	16	32
80°C 30 min	16	32
100°C 30 min	16	32

FBS, fetal bovine serum.

The effect of heat on the antibacterial activity of MR-22 was investigated by heating test. However, the antimicrobial activity of MR-22 was heat-stable, the antibacterial activity of MR-22 remained unchanged after this treatment. Unfortunately, trypsin completely eliminated the antimicrobial activity of MR-22. The results revealed a decline in the antibacterial activity of MR-22 against *E.coli* ATCC 25922, registering at (28.18 ± 1.10)% after 2 hours and declining further to (5.79 ± 0.35)% after 6 hours. Similar results were observed for MR-22 against *E.coli* E19, registering at (22.15 ± 2.47)% after 2 hours and declining further to (7.23 ± 0.87)% after 6 hours (**Supplementary Table 1**).

### 3.5 Hemolysis and cytotoxicity of MR-22

MR-22 concentrations from 2 to 256 µg/mL were used to further detect its hemolysis and cytotoxicity activity toward human red blood cells, human liver cells L-O2 and renal tubular epithelial cells HK-2. The cytotoxicity results showed that the cell viability of L-O2 cells treated with various concentrations of MR-22 was consistently above 100% (**Figure 2E**). However, the cell viability of HK-2 cells was lowest after treatment with 256 μg/mL of MR-22, with a value of 75.60%. The hemolysis results (**Figure 2F**) shown most cells were found to be intact, which indicated that MR-22 displayed low hemolytic activities against human erythrocytes. These findings suggest that MR-22 exhibits a favorable safety profile.

### 3.6 Antibacterial mechanism

**Figure 3:**
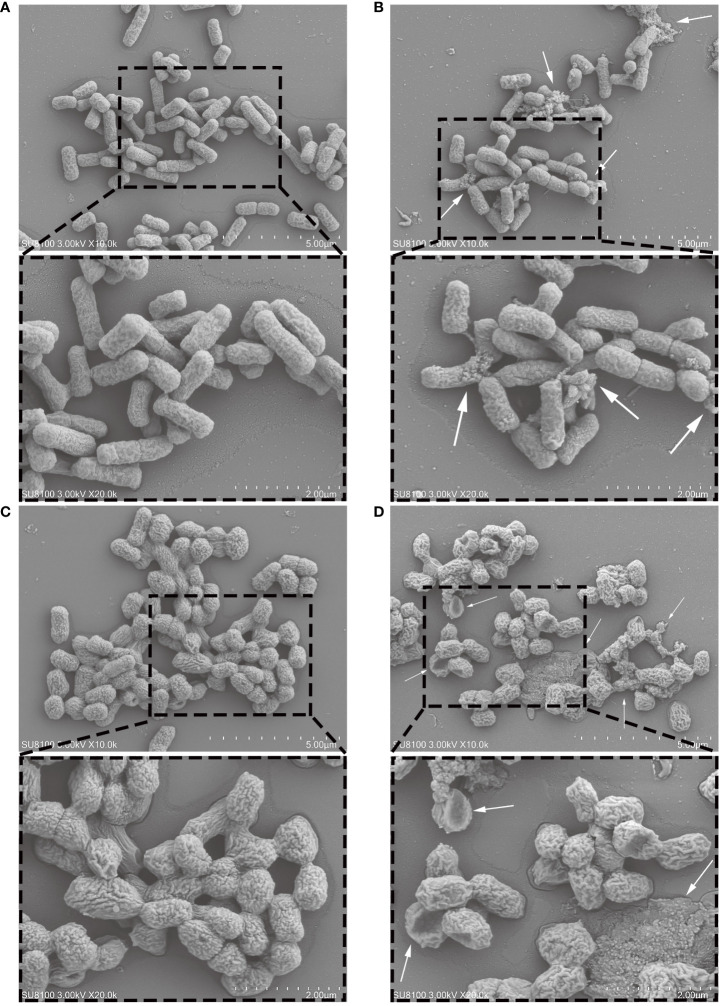
Scanning electron microscopy images of *E. coli* exposed and unexposed by MR-22. **(A)** ATCC 25922 treated without MR-22. **(B)** ATCC 25922 treated with MR-22. **(C)** E19 treated without MR-22. **(D)** E19 treated with MR-22.

The scanning electron microscopy (SEM) images showed normal *E. coli* was uniform rod-shaped with a smooth surface. And treated with MR-22 at their respective MICs for 2 h had a severe damage and ruptures (**Figure 3**).

**Figure 4:**
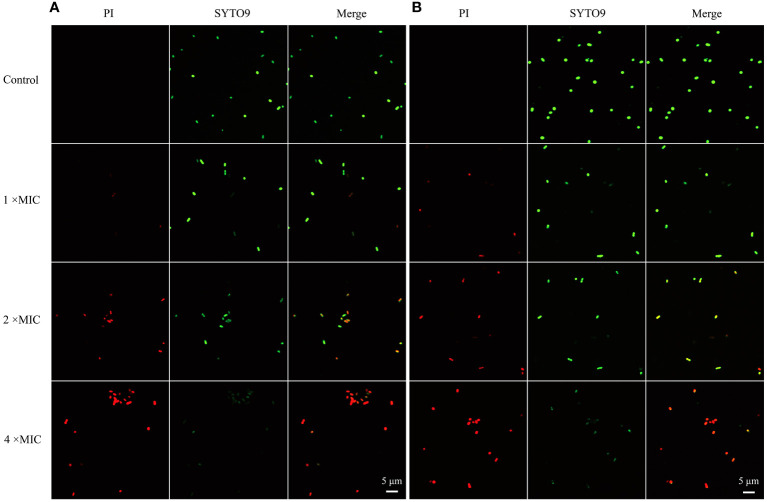
Confocal laser scanning microscopy images of *E. coli* exposed and unexposed by MR-22. **(A)** ATCC25922. **(B)** E19. The control was treated without MR-22.

To probe the mode of antibacterial action of MR-22, PI and SYTO9 nucleic acid stains were used to determine the effect of MR-22 on the integrity of the bacteria cell membrane by CLSM. PI is a red nucleic acid-binding dye that penetrates only cells with damaged membranes and STOY9 is a green fluorescent nucleic acid stain that can stain both live and dead bacteria. As shown in **Figure 4**, after 1 h untreated *E. coli* cells were stained with STOY9 but not stained with PI, indicating that the majority of cells were alive. In contrast, the number and intensity of red fluorescent spots increased in a concentration-dependent manner following the treatment with MR-22 at concentrations of 1, 2, 4 × MIC. These data demonstrating that MR-22 was able to disrupt the bacterial cell membranes, and rupture increased with the increase of peptide concentration.

**Figure 5:**
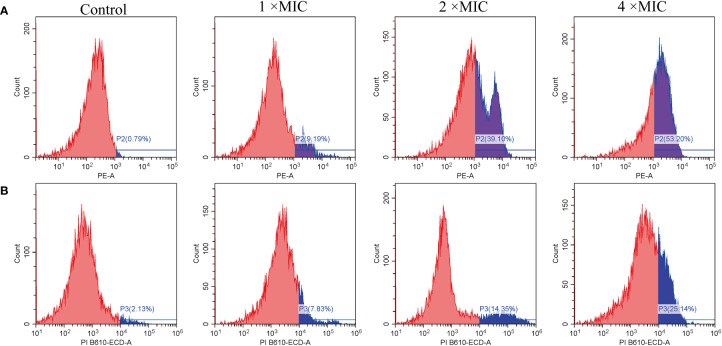
MR-22 induced membrane permeabilization of *E. coli* ATCC 25922 **(A)** and E19 **(B)**. The fluorescence intensity of propidium iodide (PI) in *E. coli* treated with different concentrations of MR-22 for 1 h was measured by flow cytometry.

PI also used as a probe to study the integrity of cell membranes(Sautrey et al., 2016). When cells are damaged, PI can penetrate the membrane and intercalate with DNA. In contrast, intact cells prevent PI from entering (Thulshan Jayathilaka et al., 2021). As shown in **Figure 5A**, untreated *E. coli* cells in the ATCC 25922 group displayed a low percentage of PI fluorescence signals, at only 0.79%. In contrast, cells treated with MR-22 at concentrations of 1, 2, 4 × MIC for 1 h demonstrated PI fluorescence signals of 9.19%, 39.10%, and 53.20%, respectively. In the *E. coli* E19 group, the percentage of PI-positive cells was 2.13% in the absence of MR-22 treatment. Following treatment with MR-22 at concentrations of 1, 2, 4 × MIC for 1 h, the percentage of PI-positive cells increased to 7.83%, 14.35%, and 25.14%, respectively (**Figure 5B**). These data collectively indicated that MR-22 damaged the integrity and improved the membrane permeability of bacterial membranes.

### 3.7 MR-22 promotes the production of ROS in *E. coli*

**Figure 6:**
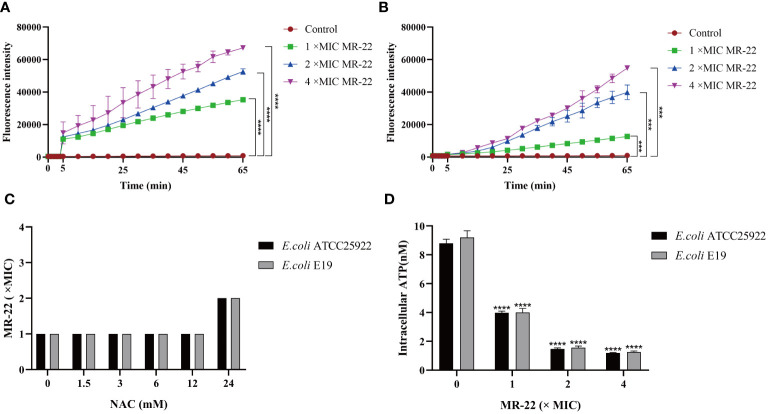
MR-22 triggers the production of ROS and decreased levels of intracellular ATP in *E. coli*. **(A, B)** MR-22 induces the production of ROS in both *E. coli* ATCC 25922 and E19. **(C)** Exogenous supplementation of NAC resulted in a decrease in the antibacterial activity of MR-22 against *E. coli*. **(D)** Assessment of the effect of MR-22 on intracellular ATP concentrations in *E. coli* ^***^ *P<*0.005 and ^****^ *P*<0.0001, compared with control.

Reactive oxygen species (ROS) play an important role in bacteria death. As shown in **Figures 6A, B**, ROS accumulation in bacteria increased significantly after the treatment MR-22. Furthermore, the addition of the antioxidant N-acetyl-cysteine (NAC) at a concentration of 24 mM reduced the production of ROS, as shown in **Figure 6C**. These findings show that ROS plays a key role in the sterilization of antibacterial peptides.

### 3.8 Assessing the impact of MR-22 on intracellular ATP in *E. coli*

The intracellular concentration of adenosine triphosphate (ATP) was measured to demonstrate the damage of energy metabolism. In this study, treatment with series concentrations of MR-22 on in *E. coli*. As shown in **Figure 6D**, comparing to the control group, ATP concentration was significantly reduced on both treatment groups of *E. coli*, and this inhibitory effect of MR-22 was dose-dependent.

### 3.9 *In vivo* antibacterial activity

**Figure 7:**
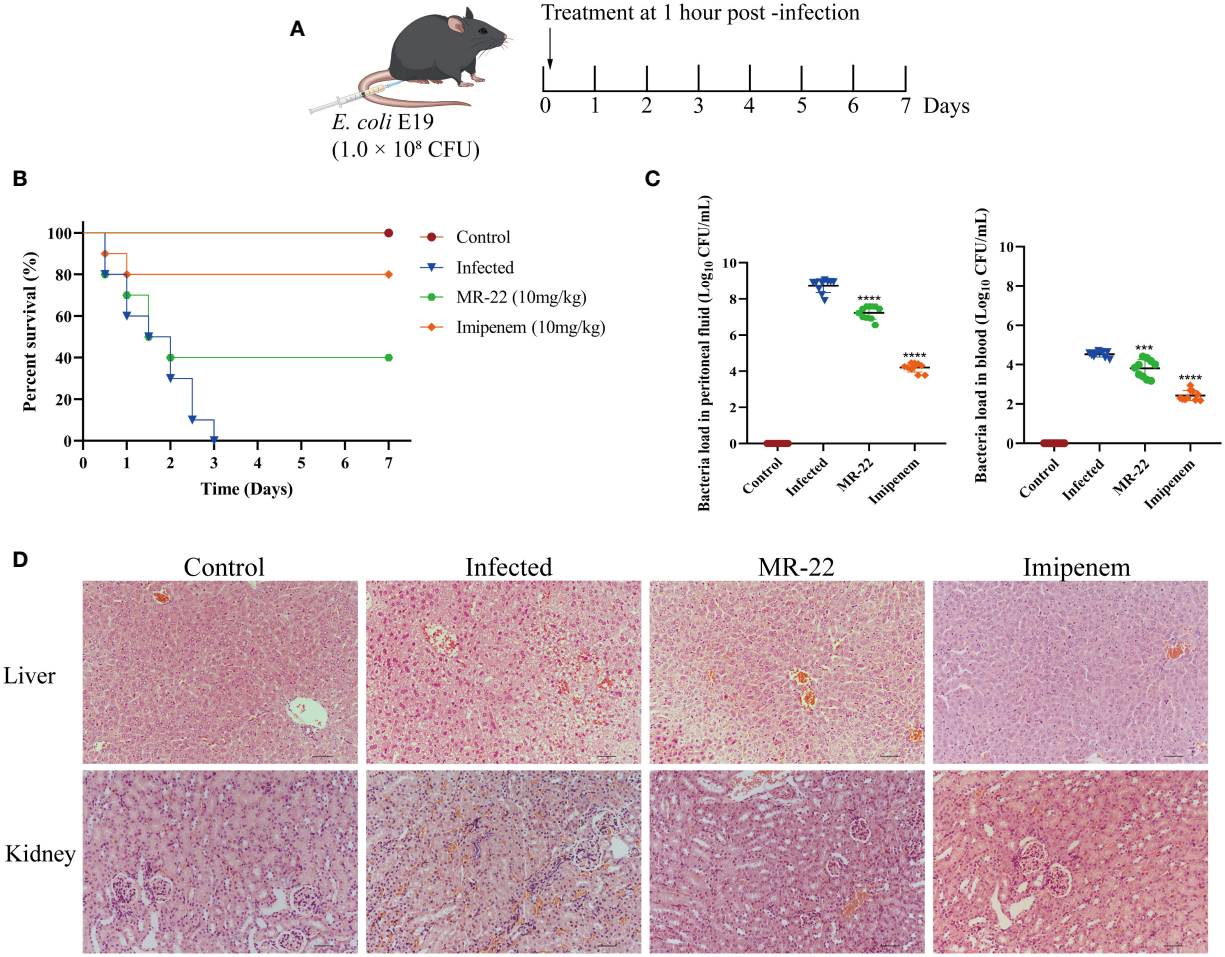
*In vivo* efficacy of MR-22 for against *E. coli* infection in the mice peritonitis model. **(A)** Scheme of the experimental protocol for the mice peritonitis model. **(B)** Survival curves of mice with E19-induced peritonitis after have been treated with MR-22. **(C)** Mice treated with MR-22 exhibit a decrease in bacterial loads in the peritoneal fluid (Compared with infection group, *** denotes *P* < 0.005 and **** denotes *P* < 0.001). **(D)** Pathological changes of liver and kidney in mice after bacterial challenge. Original Magnification, × 20.

Given that MR-22 can inhibited *E. coli*, we assessed the efficacy of MR-22 in mice model (**Figure 7A**). In C57BL/6J mice model, the 72-h survival rate was 0% in the infected group. The survival rate of the control group was 100%, and that of the MR-22 group was 40%. Therefore, administration of MR-22 at a dose of 10 mg/kg improved the survival rate of the mice (**Figure 7B**).

To further evaluate the effect of MR-22, we measured the bacterial load in mice peritoneal lavage fluid. Ten mice were used for each treatment group, and all mice received sterile PBS or 10 mg/kg of MR-22 or imipenem after 1 h of bacterial infection. After 36 h, compared to the control group with a bacterial count of 8.73 ± 0.38 Log_10_CFU/mL, the bacterial content in the peritoneal lavage fluid of peritonitis mice treated with MR-22 decreased to 7.23 ± 0.36 Log_10_CFU/mL (*P*<0.0001), while the bacterial content in the peritoneal lavage fluid of peritonitis mice treated with imipenem decreased to 4.20 ± 0.25 Log_10_CFU/mL (*P*<0.0001) (**Figure 7C**). Histopathology analysis showed that MR-22 had a therapeutic effect on peritonitis in mice (**Figure 7D**). There was severe damage in liver and kidney, characterized by bleeding, cell swelling and degeneration, and loose cytoplasm with visible cavities. However, few inflammatory symptoms of livers and kidneys in the MR-22 group. These suggested that MR-22 inhibited bacterial *in vivo*.

## 4 Discussion

Drug-resistance bacteria have become important pathogens causing hospital-acquired infections. These bacteria significantly reduced the efficacy of antibiotics, whereas developing new therapeutic drugs has become an urgent priority (de Kraker et al., 2016; Morrison and Zembower, 2020). AMPs as antibiotic preparations is an innovative approach have been used in the treatment of drug-resistant bacteria (Zhang et al., 2020).

In this study, we investigated the potent *in vitro* antibacterial activity of MR-22 against *E. coli* (**Table 2**). The MIC and MBC results showed that MR-22 inhibited *E. coli* in a dose-dependent. Effective antimicrobial peptides require rapid bactericidal action, emphasizing the importance of understanding the time-dependent changes in their antimicrobial activity. The growth curve showed that MR-22 significantly inhibited the growth of *E. coli* (**Figures 2A, B**), the bactericidal kinetics (**Figures 2C, D**) revealed a dose-dependent of MR-22. These findings indicate that MR-22 is a potent bactericidal agent with a sustained effect over 24 h, providing valuable insights into the development of novel antimicrobial peptides.

Due to the cellular membrane being the primary target of action for most antimicrobial peptides, it is not unexpected for cytotoxicity to arise, particularly hemolytic activity and cytotoxicity (Wei et al., 2020). However, previous research by Dong et al. found that hemolytic activity and cytotoxicity are also positively correlated with the hydrophobicity of the peptide (Dong et al., 2014). Interestingly, our results demonstrated that MR-22 was less toxic on normal human liver cells (L-O2), and had a certain toxic on human renal tubular cells (HK-2) (**Figure 2E**) at 256 μg/mL, had virtually no hemolytic (**Figure 2F**). These results could be attributed to the preponderance of hydrophilic amino acids in MR-22, which have been shown to reduce its hemolytic potential. These findings provide a basis for the clinical application of MR-22 for local use.

Most AMPs are sensitive to salts and enzyme (Mai et al., 2011; Moncla et al., 2011; Mahlapuu et al., 2016). The stability of antimicrobial peptides in high salt environments limit their applicability as a new therapeutic option (Chu et al., 2013). In this study, variations in the MICs values of MR-22 were noted in response to alterations in the presence of salts or serum within the environment. However, it’s noteworthy that despite antimicrobial activity changes in salts or serum, MR-22 retains its antimicrobial activity against *E. coli*. (**Table 3**). Serum stability analysis revealed that MR-22’s antimicrobial activity was suppressed in mice serum, which probably because the interaction between albumin and peptide in serum (Tang et al., 2021). When exposure to trypsin, the activity of MR-22 decreased, possibly due to the high specificity of trypsin to arginine and lysine residues (Arias et al., 2018). Similarly, Seo et al. documented a substantial decrease in the activity of SJGAP subsequent to trypsin treatment (Seo et al., 2014).

So far, the precise mechanism underlying the antibacterial action of AMPs remains elusive, with the prevailing hypothesis positing their interaction with the membrane (Mba and Nweze, 2022). We found that MR-22 can disturb the membrane fluidity and destroy the bacteria membrane structure (**Figures 3**–**5**).

The accumulation of ROS in a cell destroys various cellular components, such as proteins and lipids, thus disrupting ATP synthesis. In the present study, ROS significantly increased while ATP decreased in bacteria treated with MR-22 (**Figure 6**) suggesting that oxidative stress caused by ROS ultimately induce cell damage of *E. coli*. This phenomenon aligns with prior observations indicating the essential role of endogenous ROS in the bactericidal activity of antimicrobial agents (Aribisala and Sabiu, 2022). Therefore, MR-22 caused bacteria death by damaging the cell membrane, increasing ROS and decreasing ATP in *E. coli*.

To investigate the *in vivo* therapeutic effect of MR-22, experiments were conducted on mice with peritoneal infection caused by MDR *E. coli*. The results of the *in vivo* experiments demonstrated that MR-22 effectively reduced the bacterial burden in the peritoneum and blood of the mice, consequently lowering the mortality rate. Additionally, MR-22 demonstrated a good protective effect on the pathology of affected organs, underscoring its potential as a promising therapeutic agent for abdominal infections in mice.

**Figure 8:**
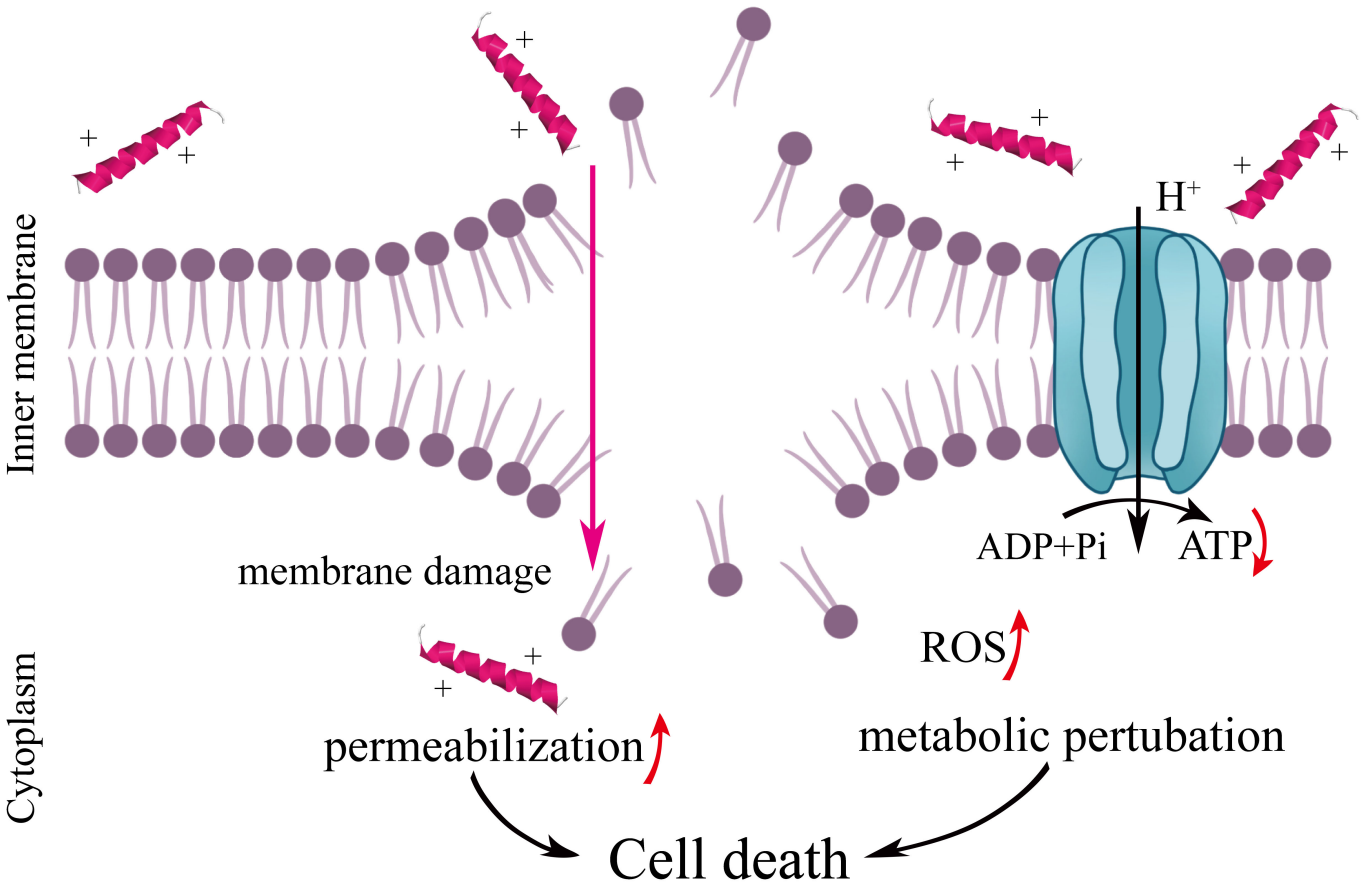
Schematic representation for the mechanism of action of MR-22 against *E. coli*. It is plausible that MR22 exerts its bactericidal effects against *E. coli* by disrupting the structural integrity of the cell membrane, resulting in membrane dysfunction, the accumulation of reactive oxygen species, perturbations in energy metabolism, and subsequent derangements in bacterial physiological processes. These cascading events culminate in the demise of *E. coli*.

In conclusion, this study reveals the bactericidal mechanism of MR-22 (**Figure 8**). Our results show that MR-22 damages the integrity of the membranes by stimulating ROS and inducing ATP decreasing in drug-resistance *E. coli*. This shows that MR-22 exhibits significant inhibitory effects on *E. coli*. Thus, this study provides possible targets for MR-22 against *E. coli* and a theoretical basis for the antibacterial activity of antimicrobial peptides.

## Data availability statement

The original contributions presented in the study are included in the article/**Supplementary Material**. Further inquiries can be directed to the corresponding author.

## Ethics statement

The studies involving humans were approved by Guiyang Hospital of Guizhou Aviation Industry Group. The studies were conducted in accordance with the local legislation and institutional requirements. The participants provided their written informed consent to participate in this study. The animal study was approved by Institutional Animal Care and Use Committee of Guizhou Medical University. The study was conducted in accordance with the local legislation and institutional requirements.

## Author contributions

CT: Writing – original draft, Writing – review & editing. NZ: Writing – original draft, Writing – review & editing. LY: Writing – review & editing. FL: Writing – review & editing. RC: Writing – review & editing. YZ: Writing – review & editing. JP: Writing – review & editing. GG: Writing – original draft, Writing – review & editing.
