# Supplementary material for: The antibacterial activity and mechanism of a novel peptide MR-22 against multidrug-resistant Escherichia coli
Source: Front Cell Infect Microbiol. 2024 Jan 24;14:1334378. doi: 10.3389/fcimb.2024.1334378 (PMC10847306; doi:10.3389/fcimb.2024.1334378)
Supplement: Supplementary file 1 [file DataSheet_1.docx]

Supplementary Material

The antibacterial activity and mechanism of a novel peptide MR-22 against Multidrug-Resistant *Escherichia coli*

Chunren Tian^1,2,3,†^, Na Zhao^3,4,†^, Longbing Yang^1^, Fei Lin^1^, Ruxia Cai^1^,Yong Zhang^1^, Jian Peng^1^ , Guo Guo^1,3,4*^

^1^School of Basic Medical Sciences, The Key and Characteristic Laboratory of Modern Pathogen Biology, Guizhou Medical University, Guiyang, People’s Republic of China

^2^Clinical Laboratory, Guiyang Hospital of Guizhou Aviation Industry Group, Guiyang, People’s Republic of China

^3^Key Laboratory of Environmental Pollution Monitoring and Disease Control, Ministry of Education, Guizhou Medical University, Guiyang, People’s Republic of China

^4^Translational Medicine Research Center, Guizhou Medical University, Guiyang, People’s Republic of China

*** Correspondence:**

Guo Guo

guoguojsc@163.com

†These authors share first authorship


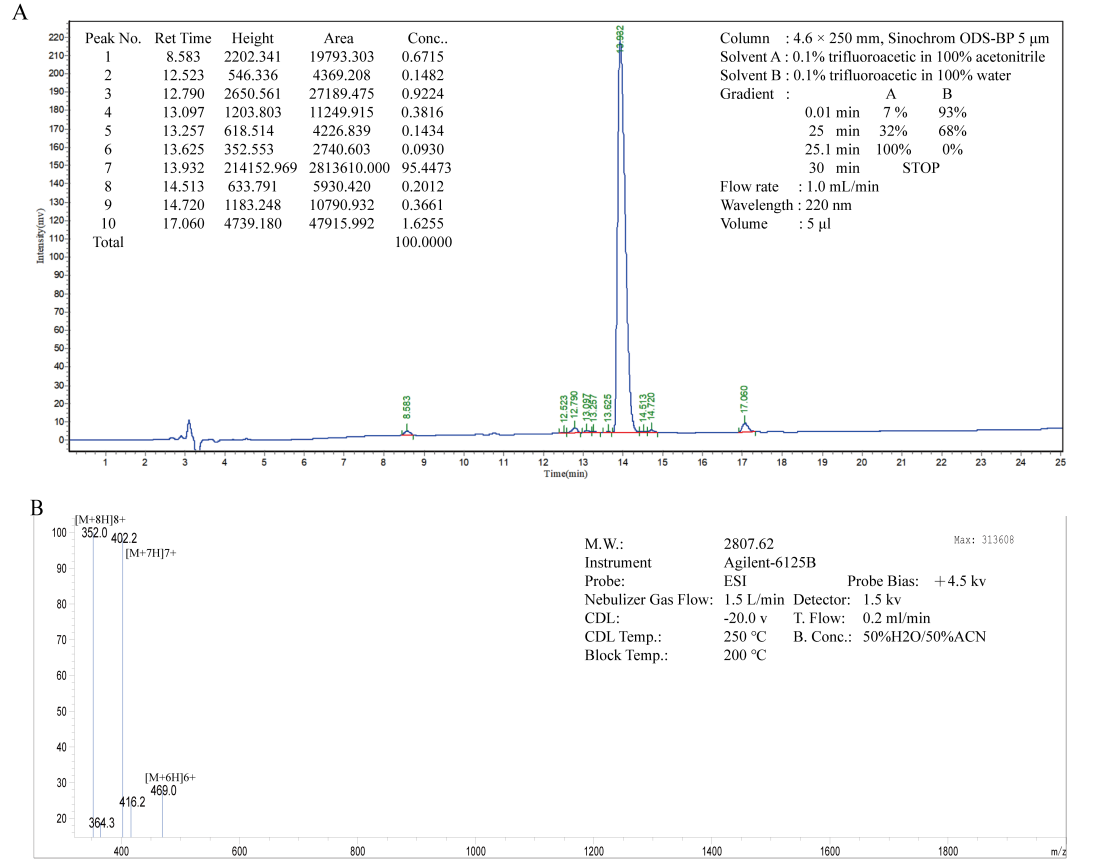


**Supplementary Figure 1.** Characterization of MR-22. (A) purification of MR-22. (B) electrospray ionization (ESI) mass spectrometer of MR-22.

**Supplementary Table 1.** Antibacterial activity of MR-22 after incubation with trypsin.

| Time (h) | Rate of inhibition（%） | |
| --- | --- | --- |
|  | *E. coli* ATCC 25922 | *E. coli* E19 |
| Control  2  4  6  8  10  12 | 100  28.18 ± 1.10  22.32 ± 1.71  5.79 ± 0.35  3.40 ± 0.26  1.85 ± 0.18  1.33 ± 0.22 | 100  22.15 ± 2.47  17.74 ± 2.88  7.23 ± 0.87  5.41 ± 1.20  4.49 ± 1.54  0.64 ± 0.46 |
